# Evaluating Proton Versus Photon Therapy: A Call for Nuanced Decision‐Making

**DOI:** 10.1002/jmrs.855

**Published:** 2025-01-10

**Authors:** Peter Gorayski, Hien Le, Frank Saran

**Affiliations:** ^1^ Royal Adelaide Hospital Adelaide South Australia Australia; ^2^ South Australia Health and Medical Research Institute Adelaide South Australia Australia; ^3^ Australian Bragg Centre for Proton Therapy and Research Adelaide South Australia Australia; ^4^ Allied Health and Human Performance Academic Unit University of South Australia Adelaide South Australia Australia

## Abstract

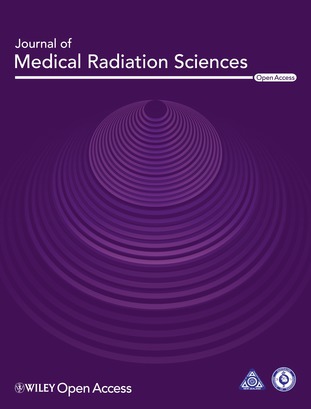

We read with interest the article “Is proton beam therapy always better than photon irradiation? Lessons from two cases,” by Michelle Li and colleagues, which addresses the complexities in selecting the optimal treatment modality for patients suitable for proton beam therapy (PBT). While we commend their balanced presentation of the risks and benefits associated with both proton and photon irradiation, we respectfully disagree with the conclusion that their data unequivocally favour photon radiation therapy (XRT) over PBT in these cases.

PBT, with its distinct physical properties, typically reduces integral dose exposure and minimises radiation to a greater number of organs at risk (OAR) compared to XRT, as shown in dose–volume histogram (DVH) studies [1, 2, 3]. Numerous published studies and international expert consensus recognise PBT as the preferred treatment for children and young adults with primary central nervous system (CNS) tumours in many healthcare systems [4, 5]. However, the two cases presented by Li et al. [6] highlight that selecting the optimal therapy is a complex process, requiring a nuanced understanding of the specific benefits and limitations of both PBT and advanced photon techniques like intensity‐modulated radiation therapy (IMRT) or volumetric‐modulated arc therapy (VMAT).

In the first case, the clinical target volume (CTV) is adjacent to a critical OAR, presenting a scenario where PBT cannot achieve the differential gradient index required to spare the OAR effectively. The second case illustrates the difficulty in generating competitive plans for very small CTVs using world‐class relocatable stereotactic treatment set‐ups, as PBT requires a robust planning algorithm with a minimal margin of 2–3 mm, leading to a relatively larger high‐dose envelope compared to stereotactic radiosurgery (SRS) set‐ups.

The target volume definitions and planning approaches utilised by Li et al. adhere to international standards; however, we question whether these methods would yield identical conclusions if applied in other centres of excellence. In the first case, the authors employ a 10 mm GTV to CTV expansion, which could safely be reduced to 5 mm based on existing protocols [7]. Additionally, the PBT robustness planning algorithm used (3.5%/3 mm for 12 uncertainty scenarios), which might be refined to 3%/2 mm with appropriate immobilisation and image guidance. This may potentially alter the dosimetric outcomes in favour of PBT in this case.

Furthermore, the selection of hippocampal dose as the pivotal OAR differential in case 1 may not be the most appropriate surrogate for cognitive outcomes. Recent phase 2 data have used temporal lobe dose as a surrogate, correlating more closely with cognitive outcomes [8]. Using such metrics might shift the conclusions regarding the preferential radiation modality.

In case 2, the choice of a 1 mm gross tumour volume (GTV) to CTV margin for a pilocytic astrocytoma could be re‐evaluated, as many clinicians employ a 5 mm margin in accordance with recent paediatric studies [9]. The capability to deliver linear accelerator (LINAC)‐based CNS SRS with a 0.5 mm set‐up uncertainty is commendable, but not widely achievable. A more typical CTV to PTV margin is around 1 mm and adjusting these parameters could affect the risk–benefit analysis presented.

Lastly, the consideration of integral dose differences and the associated risk of secondary malignant neoplasms (SMNs) is crucial, particularly in paediatric radiation therapy [10]. The vertex partial arc used in case 1 may increase the risk of inducing SMNs by exposing larger areas of the body to low doses of radiation. Early clinical data suggest that PBT may halve the excess risk of radiation‐induced SMNs compared to XRT, although long‐term follow‐up is necessary to confirm these findings [11, 12].

The decision‐making process in radiation therapy, especially for paediatric cases, is multifaceted and should not be oversimplified to dosimetric comparisons of a single OAR. Optimal radiation therapy (RT) modality selection requires integrating multiple factors, including potential late effects such as secondary malignancies, endocrine dysfunctions, hearing loss and vision impairments. A model‐based selection process, like the one developed in the Netherlands for head and neck cancer, could enhance the comparative planning process for PBT in the future [13].

In summary, Li et al. provide important insights into selecting RT modalities, though their conclusions warrant additional consideration. Future discussions should adopt a comprehensive approach, considering all potential risks and benefits to optimise long‐term outcomes for paediatric and adolescent and young adult patients. Initiatives like the Trans‐Tasman Radiation Oncology Group (TROG) Australian Particle Therapy Clinical Quality Registry (ASPIRE) [14] provide a valuable opportunity for the Australian oncological community to contribute significantly to the evidence base, supporting well‐informed decisions regarding PBT.

## Conflicts of Interest

The authors declare no conflicts of interest.

## Data Availability

Data sharing is not applicable to this article as no new data were created or analyzed in this study.
